# Real-Time Evaluation of Cerebral Autoregulation Based on Near-Infrared Spectroscopy to Predict Clinical Outcome after Bypass Surgery in Moyamoya Disease

**DOI:** 10.1155/2022/3091660

**Published:** 2022-08-21

**Authors:** Junmo Kim, Eun-Jin Ha, Hee-Soo Kim, Eun-Young Park, Hyung-Chul Lee, Yoon-Hee Choo, Youngbo Shim, Kwangsoo Kim, Keewon Kim, Seung-Bo Lee

**Affiliations:** ^1^Interdisciplinary Program in Bioengineering, Seoul National University, Seoul 08826, Republic of Korea; ^2^Department of Critical Care Medicine, Seoul National University College of Medicine, Seoul National University Hospital, Seoul 03080, Republic of Korea; ^3^Department of Anesthesiology and Pain Medicine, Seoul National University College of Medicine, Seoul National University Hospital, Seoul 03080, Republic of Korea; ^4^Department of Neurosurgery, Seoul St. Mary's Hospital, College of Medicine, The Catholic University of Korea, Seoul 06591, Republic of Korea; ^5^Transdisciplinary Department of Medicine and Advanced Technology, Seoul National University Hospital, Seoul 03080, Republic of Korea; ^6^Department of Medicine, College of Medicine, Seoul National University, Seoul 03080, Republic of Korea; ^7^Department of Rehabilitation Medicine, Seoul National University College of Medicine, Seoul National University Hospital, Seoul 03080, Republic of Korea; ^8^Department of Medical Informatics, Keimyung University School of Medicine, Daegu 42403, Republic of Korea

## Abstract

Impaired cerebral autoregulation (CA) can cause negative outcomes in neurological conditions. Real-time CA monitoring can predict and thereby help prevent postoperative complications for neurosurgery patients, especially those suffering from moyamoya disease (MMD). We applied the concept of moving average to the correlation between mean arterial blood pressure (MBP) and cerebral oxygen saturation (S_C_O_2_) to monitor CA in real time, revealing optimal window size for the moving average. The experiment was conducted with 68 surgical vital-sign records containing MBP and S_C_O_2_. To evaluate CA, the cerebral oximetry index (COx) and coherence obtained from transfer function analysis (TFA) were calculated and compared between patients with postoperative infarction and those who without. For real-time monitoring, the moving average was applied to COx and coherence to determine the differences between groups, and the optimal moving-average window size was identified. The average COx and coherence within the very-low-frequency (VLF) range (0.02-0.07 Hz) during the entire surgery were significantly different between the groups (COx: AUROC = 0.78, *p* = 0.003; coherence: AUROC = 0.69, *p* = 0.029). For the case of real-time monitoring, COx showed a reasonable performance (AUROC > 0.74) with moving-average window sizes larger than 30 minutes. Coherence showed an AUROC > 0.7 for time windows of up to 60 minutes; however, for windows larger than this threshold, the performance became unstable. With an appropriate window size, COx showed stable performance as a predictor of postoperative infarction in MMD patients.

## 1. Introduction

Cerebral autoregulation (CA) is the physiological mechanism that maintains constant blood flow despite changes in cerebral perfusion pressure (CPP) [1]. As long as CA remains intact, the brain can protect itself against excessively high or low blood flow regardless of CPP. Impaired CA is strongly associated with negative outcomes in a variety of neurological conditions such as traumatic brain injury, intracranial hemorrhage, and cerebral infarction [2–4].

One of the cerebrovascular diseases related to CA is moyamoya disease (MMD). MMD is a clinical entity characterized by stenosis and occlusion of the terminal internal carotid arteries, together with the development of collateral networks, which seem to provide an alternative route for cerebral perfusion [5]. MMD is difficult to treat because the cause of blood vessel blockage is not clear; however, bypass is performed to prevent cerebral infarction. Although the etiology of MMD is still unknown, several studies have shown the correlation between MMD and impaired CA [6, 7]. Among MMD patients, the occurrence of infarction is a major form of neurological damage that is closely related to impaired CA [8].

Noninvasive parameters that are widely used to evaluate CA include the mean velocity index (Mx) and the cerebral oximetry index (COx). Mx indicates the correlation of cerebral blood flow velocity (CBFV) with mean arterial blood pressure (MBP) and is calculated based on the Pearson correlation coefficient (PCC) [9]. Measurement of CBFV requires application of a Doppler ultrasound probe to a specific window in the skull, which cannot be maintained for prolonged time in practice. COx is calculated as the PCC between MBP and cerebral oxygen saturation (S_C_O_2_), and S_C_O_2_ can be continuously measured using near-infrared spectroscopy (NIRS) sensors [10, 11]. In addition to Mx and COx, some studies use frequency-domain approaches with transfer function analysis (TFA), yielding indicators for evaluating CA such as coherence, gain, and phase [12]. All these indicators are exquisitely developed but are not practical for real-time monitoring; CBFV for Mx is highly sensitive and cannot be collected for prolonged time. Additionally, COx and TFA indicators hardly obtain clinical rationale when real-time monitoring because of their fluctuation.

This study proposes a novel CA monitoring method to predict postoperative infarction in MMD surgical patients by analyzing MBP and S_C_O_2_ in “real time.” Applying the concept of moving average to indicators obtained from MBP and S_C_O_2_ in real time, we defined the optimal moving-average window size for COx and coherence, one of the autoregulatory parameters obtained by TFA, and the performance was statistically significant.

## 2. Material and Methods

### 2.1. Patient Inclusion and Data Acquisition

This study was approved by Seoul National University College of Medicine/Seoul National University Hospital Institutional Review Board (IRB approval No. 2102-113-1197) in agreement with the Declaration of Helsinki, Korean Bioethics and Safety Act (Law No. 16372), and Human Research Protection Program-Standard Operating Procedure of Seoul National University Hospital. Patient consent was not required by our IRB for this database due to the retrospective nature of the study and the lack of patient interaction.

During the construction of the VitalDB, vital signs from various medical devices were collected prospectively using the Vital Recorder program [13]. Analog-to-digital converters (SNUADC, VitalLab, Seoul, Korea) were used to obtain 500 Hz MBP waveform signals from the analog output port of the patient monitor (Tram module of Solar™ 8000 patient monitor, GE Healthcare, Wauwatosa, WI, USA), and S_C_O_2_ data were obtained from an INVOS oximeter (Medtronic, Minneapolis, MN) at 5-second intervals. Two INVOS sensors were attached to the forehead above both eyebrows immediately after a patient entered the operating room and removed shortly before the patient left the operating room.

From the registry, the 68 records of adult (>18 years) MMD patients aged 22 to 65 years who underwent bypass surgery from February 2018 to June 2020 were collected. Improperly collected signals due to dislodged sensors or mechanical defects were excluded.

### 2.2. NIRS-Based Autoregulation Monitoring

MBP was collected in a 500 Hz waveform, and NIRS S_C_O_2_ was collected as a numeric variable at 0.2 Hz. For the NIRS signal, cubic spline interpolation was applied to convert the numeric type to the waveform type [14]. To remove outliers due to artifacts, a Hampel filter was applied, and intervals unmeasured due to dislodged sensors were removed, including 10 seconds before and after [15]. Data with a length less than 10% of the original length after removal were considered impaired data. The signals were filtered to a nonoverlapping 10-second average value, which is equivalent to using a moving-average filter with a 10-second time window and resampling at 0.1 Hz. This method reduces the effect of high-frequency components due to breathing and pulse waveforms [16, 17]. If NIRS signals from both sides were damaged, the record was excluded from the study, and if only one signal was damaged, the other one was used for the analysis. If both signals were not damaged, we used the mean values of those signals; however, the intervals in which the difference in values between the two signals was too high (>30%) were removed. To investigate the difference between the signals from the operated and contralateral sides, we performed the Mann–Whitney *U* test and there was no difference in S_C_O_2_ with a *p* value larger than 0.15.

COx was calculated as the PCC between MBP and S_C_O_2_ every 10 seconds. When CA is intact, COx approaches zero or negative while impaired CA is indicated by high positive COx. TFA was performed for each pair of signals using the classical fast Fourier transform approach combined with Welch's method [18]. Each 20-minute epoch was divided into 5 segments overlapping by 75% for spectral estimation. The transfer function (TF) gain, phase, and coherence derived from TFA were calculated using the cross-power spectral density between the input (MBP) and the output (S_C_O_2_) and the respective autopower spectral densities of MBP and S_C_O_2_. TF gain, phase, and coherence were averaged over the frequency bands of 0.02-0.07 Hz (very low frequency (VLF)), 0.07-0.2 Hz (low frequency (LF)), and 0.2-0.5 Hz (high frequency (HF)).

### 2.3. Clinical Outcomes

The clinical outcomes after surgery were evaluated to validate the clinical utility of CA parameters. The occurrence of postoperative infarction, hyperperfusion, and intracranial hemorrhage (ICH) was reviewed, as were the patients' Glasgow Outcome Scale (GOS) scores [19]. Infarction or ICH was confirmed by magnetic resonance imaging (MRI). All the patients received a recent preoperative MRI, and the patients with neurological symptoms after the surgery received postoperative MRI. Postoperative infarction was recorded if there were new lesions by comparing preoperative MRI with postoperative MRI on the operated hemisphere. Hyperperfusion was clinically suspected and confirmed with MRI when neurological symptoms from hyperperfusion did not resolve with empirical management [20]. GOS was routinely evaluated at discharge. In this study, the primary outcome was postoperative infarction because it resulted in definite neurological deficits with evidence from MRI, and postoperative hyperperfusion was the secondary outcome.

### 2.4. Statistical Analysis

The comparison of characteristics between the infarction group and the noninfarction group were investigated by calculating *p* values using Fisher's exact test for categorical variables and the Mann–Whitney *U* test for continuous variables. To find and validate the optimal window size of moving average for COx and TF coherence within the VLF range, area under the receiver operating characteristic curve (AUROC) values for infarction status were calculated according to various window sizes from real time to 480 minutes. AUROC values calculated with window sizes of 10, 20, 30, and 60 minutes were compared to AUROC calculated with entirely averaged value, and the comparison of AUROC values were performed by the method of Hanley and McNeil [21]. Statistical significance was set at *α* = 0.05. All signal processing, numerical computation, and statistical analysis were performed using the Python programming language (version 3.6.9).

## 3. Results

A summary of the characteristics of the patients and raw signals during surgery is shown in Table 1. There was no significant difference between the groups except in the number of unilateral MMDs, modified Rankin scale (mRS) score after discharge, and postoperative GOS. Raw vital signals, such as MBP, S_C_O_2_, and EtCO_2_, were also not significantly different. A comparison of autoregulatory parameters between the infarction group and the noninfarction group is shown in Table 2. There were significant intergroup differences in COx, coherence within the VLF and LF ranges, and in gain within the LF range. Both COx and coherence were higher in the infarction group. Gains within all frequency ranges were also slightly higher in the infarction group. Phases did not show any pattern between the infarction and noninfarction groups.

**Figure 1:**
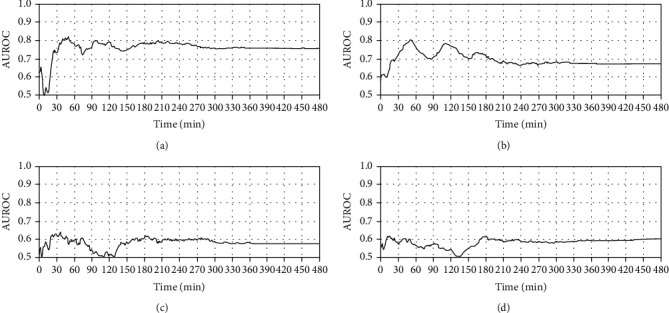
AUROC according to cumulative moving-average window size. (a) COx for infarction status. (b) Coherence for infarction status. (c) COx for hyperperfusion status. (d) Coherence for hyperperfusion status.

**Figure 2:**
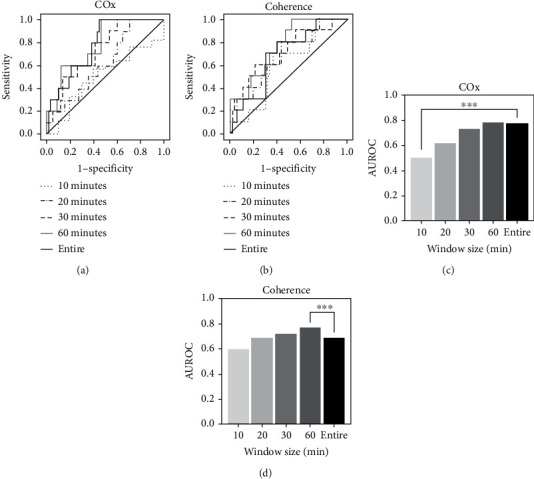
ROC curve and AUROC for infarction status according to cumulative moving-average intervals. (a, c) ROC curve and AUROC for infarction status according to cumulative mean calculation interval of COx. (b, d) The corresponding information for coherence. Two-sided statistical comparisons were performed using the Hanley-McNeil test. ∗∗∗ denotes a *p* value < 0.1, and all other values have a *p* value greater than 0.1.

To find an appropriate moving-average window size, the AUROC values for infarction and hyperperfusion status were calculated according to the cumulative moving-average window size as shown in Figure 1. In terms of infarction status, first, with a window size of 30 minutes, the cumulative mean COx showed an AUROC of approximately 0.74 to predict infarction, and the AUROC was even greater than 0.8 with a 40-minute window size, and at the end of surgery, the AUROC was approximately 0.77. The cumulative mean coherence with the 30-minute window size shows an AUROC of greater than 0.7 and oscillates between 0.7 and 0.8 until approximately 150 minutes. As the window size exceeds 150 minutes, the AUROC score decreases and remains at approximately 0.68. In terms of hyperperfusion status, both COx and coherence remained at approximately 0.6. The receiver operating characteristic curve and the bar graph comparing the AUROC measured over the entire surgery with the cumulative mean cases are shown in Figure 2. To compare the result over the entire surgery with the result of moving-average application, the method of Hanley and McNeil was applied, and for both COx and coherence, there was no significant difference in AUROC compared to the result of the entire surgery, regardless of the applied window size.

## 4. Discussion

In this study cohort, the AUROC for predicting postoperative infarction was 0.78 when COx was calculated over the entire length of the surgery, demonstrating that patients with impaired CA are at postoperative risk compared with those without impaired CA. To prevent postoperative side effects such as infarction, it is clinically important to monitor and control CA in real time during surgery. This study sought the appropriate method to monitor CA in real time by applying the concept of moving average in a cohort of patients with MMD, discovering the optimal window size for the moving average of COx and coherence.

**Figure 3:**
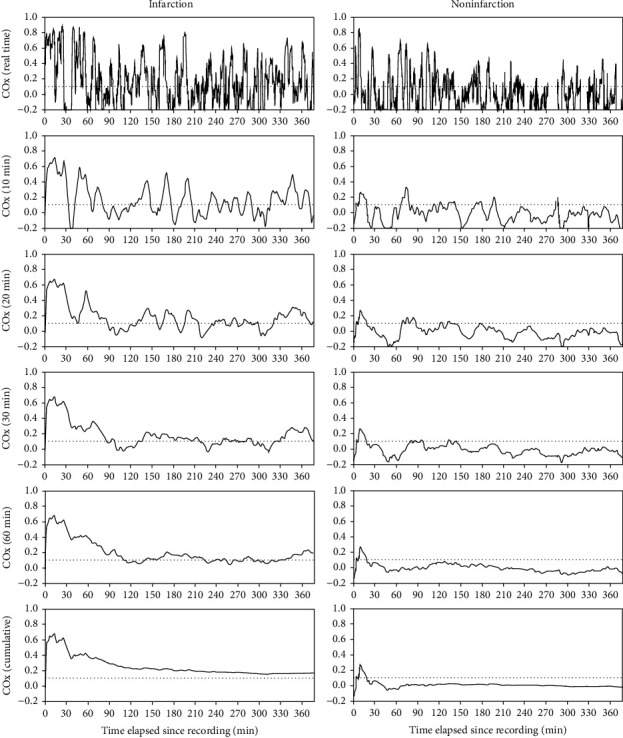
Illustrative cases of the moving average of COx with varying window sizes. The graphs on the left are from a patient who developed postoperative infarction, and the graphs on the right are from a patient who did not develop postoperative infarction. The graphs at the top show real-time COx, and the graphs at the bottom show the cumulative moving average of COx. The rest are moving-average graphs over varying window sizes.

**Figure 4:**
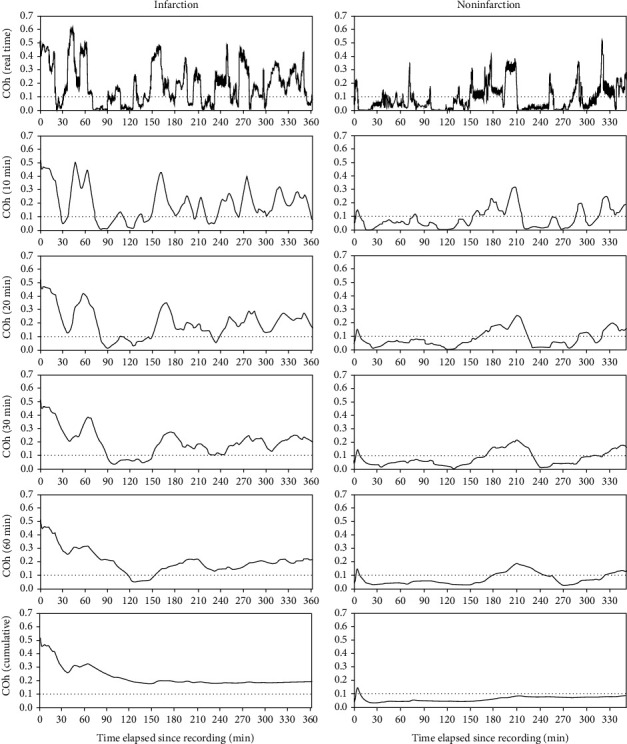
Illustrative cases of the moving average of coherence (Coh) within the VLF range with varying window sizes. The graphs on the left are from a patient who developed postoperative infarction, and the graphs on the right are from a patient who did not develop postoperative infarction. The graphs at the top show real-time coherence, and the graphs at the bottom show the cumulative moving average of coherence. The rest are moving-average graphs over varying window sizes.

Several previous studies have shown that COx is a good indicator of CA, and autoregulatory parameters from TFA, such as gain, phase, and coherence, are useful to detect impaired CA [22, 23]. Similarly, we found the intuitively plausible result that COx and coherence within the VLF range are good indicators of the risk of postoperative infarction in MMD patients, with significant differences between the infarction group and the noninfarction group. When COx and coherence were monitored in real time, however, they were similar to random signals, so the difference between groups was not clearly visible, as shown in Figures 3 and 4. While the difference between groups became clearer as the window size of the moving average increased, there are fatal drawbacks when a large window size was applied to the moving average. First, monitoring is not valid until the surgery time reached the corresponding window size. Second, when errors such as defects in the sensor or artifact entered the window, such errors are less likely to be detected and can compromise all data within the time window. Therefore, it is necessary to find an optimal window size that is sufficiently small.

The cumulative moving-average approach determined the optimal window size for COx and coherence within the VLF range to detect postoperative infarction. The window size of 30 minutes was sufficient to achieve performance above an AUROC of 0.7 for both COx and coherence. The results of the Hanley-McNeil test showed that there was no statistically significant difference between analyzing the entire surgical record and moving-average monitoring with a 30-minute window size in predicting postoperative infarction. This demonstrates that records do not need to be measured entirely to evaluate CA. Applying an appropriate moving average in CA evaluation can detect the risk of postoperative infarction in real time so that “prevention” during the surgery rather than “prediction” after the surgery is available. Meanwhile, the practical threshold was calculated as 0.1 for both COx and coherence. Maintaining the moving-average value of COx and coherence below the threshold would contribute to the prevention of postoperative infarction. For the case of postoperative hyperperfusion, neither COx nor coherence showed significant performance. In our cohort, hyperperfusion was recorded by asterial spin labeling MRI, and thus, if the hyperperfusion was controlled by medication or blood pressure regulation so that MRI examination was not required, it might not have been recorded according to decision of physician. Compared to infarction, hyperperfusion was recorded with the bias of physicians, so postoperative hyperperfusion does not appear to be detected by COx and coherence.

While COx showed stable performance as the moving-average window size increased, the instability of coherence with a large moving-average window size explains its sensitivity to signal-to-noise ratio (SNR). To perform TFA, power spectral density derived from the fast Fourier transform is needed, and as the noise intervention on signal increases, the SNR decreases and indicators obtained from TFA can be ambiguous [24]. Drugs and anesthetics used during the surgical procedure of patients with MMD can cause noise intervention on the vital signals and distortion of autoregulatory parameters obtained from TFA [25, 26]. As surgery progresses and the moving-average window size increases, more distorted values would be included into the window, and accordingly, the performance for identifying postoperative infarction appears to deteriorate. As shown in Figure 1, however, coherence also performs as much as COx with a moving-average window size between 30 and 60 minutes, which concludes that there is a delay time until surgical factors fully influence SNR. To investigate this delay time, a study on the decrease of SNR and failure of coherence monitoring under drug treatment is necessary.

Several limitations of the present study should be considered. First, since this study was limited by its small sample size (68 records) and included only patients with MMD, there could be bias in the results. Although the performance was statistically significant, this result is likely to be limited to the patients in our cohort. Second, S_C_O_2_ signal from INVOS oximeter was recorded at 0.2 Hz, which is a relatively low frequency. Thus, we were not able to perform waveform analysis using raw signals and introduced an interpolation technique assuming that the signal follows the cubic spline function. Third, all the records had information about postoperative hemorrhage and hyperperfusion, and we conducted the same experiments on them; however, the performance was not as good as that of infarction. Additionally, further experiments are needed to confirm the decrease of SNR and the failure of coherence monitoring due to surgical factors, which is a remaining research task.

## 5. Conclusion

This study proposed a novel method to monitor CA in real time for MMD patients to prevent postoperative infarction using signals from NIRS and the concept of moving average. The optimal window size for the moving average of COx and coherence was found, enabling the prevention of postoperative infarction during the surgery, rather than after the surgery.

## Figures and Tables

**Table 1:** Summary of patient characteristics and raw signals during surgery.

Variables	All (*n* = 68)	Infarction (*n* = 10)	Noninfarction (*n* = 58)	*p* value
Age (years)	39.54 ± 10.86	41.1 ± 8.73	39.28 ± 11.16	0.225
Male sex	23 (33%)	3 (30%)	20 (34%)	1.0
Operation time (hours)	6.3 ± 0.78	5.97 ± 0.64	6.35 ± 0.79	0.123
MBP (mmHg)	94.21 ± 8.44	93.16 ± 7.15	94.39 ± 8.63	0.329
S_C_O_2_ (%)	75.95 ± 7.67	73.02 ± 7.34	76.46 ± 7.61	0.077
EtCO_2_ (mmHg)	37.27 ± 1.64	37.39 ± 1.99	37.24 ± 1.57	0.329
Preoperative neurological deficit				
Duration at symptom onset (month)	0.99 ± 0.12	1.0 ± 0.0	0.98 ± 0.13	0.354
TIA	59 (86%)	10 (100%)	49 (84%)	0.336
Infarction	37 (54%)	5 (50%)	32 (55%)	1.0
Hemorrhage	14 (20%)	2 (20%)	12 (20%)	1.0
Preoperative perfusion				
ASL (MRI)				
Decreased CBF	56 (82%)	8 (80%)	48 (82%)	0.619
SPECT				
Basal hypoperfusion	43 (63%)	8 (80%)	35 (60%)	0.311
Decreased vascular reserve	64 (94%)	10 (100%)	54 (93%)	1.0
PCA involvement	22 (32%)	1 (10%)	21 (36%)	0.149
Unilateral moyamoya	10 (14%)	4 (40%)	6 (10%)	0.034
mRS score				
On admission	0.66 ± 0.85	0.4 ± 0.66	0.71 ± 0.87	0.143
Discharge	0.93 ± 1.02	1.7 ± 1.68	0.79 ± 0.78	0.036
Surgical categories				
Direct/combined bypass	67 (98%)	10 (100%)	57 (98%)	1.0
Follow-up events				
Hyperperfusion	21 (30%)	3 (30%)	18 (31%)	1.0
ICH	1 (1%)	0 (0%)	1 (1%)	1.0
Postoperative GOS score	4.79 ± 0.58	4.3 ± 1.19	4.88 ± 0.33	0.011

All data are shown as the mean ± SD and *n* (%) in the table. MBP: mean blood pressure; S_C_O_2_: cerebral oxygen saturation; EtCO_2_: end-tidal carbon dioxide concentration; TIA: transient ischemic attack; ASL: arterial spin labeling; PCA: posterior cerebral artery; mRS: modified Rankin scale; ICH: intracranial hemorrhage; GOS: Glasgow Outcome Scale.

**Table 2:** Comparison of autoregulatory parameters between the infarction group and the noninfarction group.

Variables	Infarction (*N* = 10)	Noninfarction (*N* = 58)	*p* value	AUROC
COx	0.07 ± 0.07	0.01 ± 0.08	0.003	0.78
TFA result				
*Coherence*				
VLF	0.16 ± 0.05	0.13 ± 0.05	0.029	0.69
LF	0.10 ± 0.02	0.09 ± 0.02	0.037	0.68
HF	0.09 ± 0.01	0.08 ± 0.01	0.191	0.59
*Gain*				
VLF	−18.34 ± 2.57	−19.72 ± 3.70	0.077	0.64
LF	−24.51 ± 2.31	−25.99 ± 4.37	0.049	0.67
HF	−31.11 ± 1.95	−32.26 ± 4.68	0.147	0.61
*Phase*				
VLF	0.21 ± 0.31	0.06 ± 0.48	0.140	0.61
LF	−0.23 ± 0.25	−0.11 ± 0.38	0.140	0.61
HF	0.08 ± 0.17	0.15 ± 0.22	0.125	0.62

All data are shown as the mean ± SD in the table. COx: cerebral oximetry index; TFA: transfer function analysis; VLF: very low frequency; LF: low frequency; HF: high frequency.

## Data Availability

The datasets generated during and/or analyzed during the current study are available from the corresponding author on reasonable request.

## References

[1] Paulson O., Strandgaard S., Edvinsson L. (1990). Cerebral autoregulation. *Cerebrovascular and brain metabolism reviews*.

[2] Czosnyka M., Smielewski P., Kirkpatrick P., Menon D. K., Pickard J. D. (1996). Monitoring of cerebral autoregulation in head-injured patients. *Stroke*.

[3] Jaeger M., Schuhmann M. U., Soehle M., Nagel C., Meixensberger J̈. (2007). Continuous monitoring of cerebrovascular autoregulation after subarachnoid hemorrhage by brain tissue oxygen pressure reactivity and its relation to delayed cerebral infarction. *Stroke*.

[4] Guo Z.-N., Xing Y., Wang S., Ma H., Liu J., Yang Y. (2015). Characteristics of dynamic cerebral autoregulation in cerebral small vessel disease: diffuse and sustained. *Scientific Reports*.

[5] Liming Z., Weiliang S., Jia J. (2021). Impact of blood pressure changes in cerebral blood perfusion of patients with ischemic moyamoya disease evaluated by SPECT. *Journal of Cerebral Blood Flow and Metabolism*.

[6] Kuroda S., Houkin K. (2012). Bypass surgery for moyamoya disease. *Neurologia Medico-Chirurgica (Tokyo)*.

[7] Chen J., Liu J., Duan L. (2013). Impaired dynamic cerebral autoregulation in moyamoya disease. *CNS Neuroscience & Therapeutics*.

[8] Teng C.-H., Yang I.-H., Wu M.-N., Chou P. S. (2021). Posterior reversible encephalopathy syndrome (PRES) in a patient with moyamoya disease: a case report. *Medicine*.

[9] Yam A. T., Lang E. W., Lagopoulos J. (2005). Cerebral autoregulation and ageing. *Journal of Clinical Neuroscience*.

[10] Brady K. M., Lee J. K., Kibler K. K. (2007). Continuous time-domain analysis of cerebrovascular autoregulation using near-infrared spectroscopy. *Stroke*.

[11] Sen A. N., Gopinath S. P., Robertson C. S. (2016). Clinical application of near-infrared spectroscopy in patients with traumatic brain injury: a review of the progress of the field. *Neurophotonics*.

[12] Zhang R., Zuckerman J. H., Giller C. A., Levine B. D. (1998). Transfer function analysis of dynamic cerebral autoregulation in humans. *American Journal of Physiology-Heart and Circulatory Physiology*.

[13] Lee H.-C., Jung C.-W. (2018). Vital Recorder--a free research tool for automatic recording of high- resolution time-synchronised physiological data from multiple anaesthesia devices. *Scientific Reports*.

[14] McKinley S., Levine M. (1998). Cubic spline interpolation. *College of the Redwoods*.

[15] Pearson R. K., Neuvo Y., Astola J., Gabbouj M. (2016). Generalized hampel filters. *EURASIP Journal on Advances in Signal Processing*.

[16] Brady K., Joshi B., Zweifel C. (2010). Real-time continuous monitoring of cerebral blood flow autoregulation using near-infrared spectroscopy in patients undergoing cardiopulmonary bypass. *Stroke*.

[17] Vesoulis Z. A., Liao S. M., Trivedi S. B., Ters N. E., Mathur A. M. (2016). A novel method for assessing cerebral autoregulation in preterm infants using transfer function analysis. *Pediatric Research*.

[18] Welch P. (1967). The use of fast Fourier transform for the estimation of power spectra: a method based on time averaging over short, modified periodograms. *IEEE Transactions on Audio and Electroacoustics*.

[19] Wright J., Kreutzer J. S., DeLuca J., Caplan B. (2011). Glasgow Outcome Scale. *Encyclopedia of Clinical Neuropsychology*.

[20] Yu J., Zhang J., Li J., Zhang J., Chen J. (2020). Cerebral hyperperfusion syndrome after revascularization surgery in patients with moyamoya disease: systematic review and meta-analysis. *World Neurosurgery*.

[21] Hanley J. A., McNeil B. J. (1983). A method of comparing the areas under receiver operating characteristic curves derived from the same cases. *Radiology*.

[22] Moerman A. T., Vanbiervliet V. M., van Wesemael A., Bouchez S. M., Wouters P. F., de Hert S. G. (2015). Assessment of cerebral autoregulation patterns with near-infrared spectroscopy during pharmacological-induced pressure changes. *Anesthesiology*.

[23] Tzeng Y.-C., Ainslie P. N., Cooke W. H. (2012). Assessment of cerebral autoregulation: the quandary of quantification. *American Journal of Physiology-Heart and Circulatory Physiology*.

[24] Liu X., Czosnyka M., Donnelly J. (2020). Assessment of cerebral autoregulation indices - a modelling perspective. *Scientific Reports*.

[25] Claassen J. A., Meel-van den Abeelen A. S., Simpson D. M., Panerai R. B., on behalf of the international Cerebral Autoregulation Research Network (CARNet) (2016). Transfer function analysis of dynamic cerebral autoregulation: a white paper from the International Cerebral Autoregulation Research Network. *Journal of Cerebral Blood Flow and Metabolism*.

[26] Ruesch A., Acharya D., Schmitt S., Yang J., Smith M. A., Kainerstorfer J. M. (2021). Comparison of static and dynamic cerebral autoregulation under anesthesia influence in a controlled animal model. *PLoS One*.

